# Macrophages in the pancreas: Villains by circumstances, not necessarily by actions

**DOI:** 10.1002/iid3.345

**Published:** 2020-09-03

**Authors:** Andrea F. Cruz, Rokhsareh Rohban, Farzad Esni

**Affiliations:** ^1^ Division of Pediatric General and Thoracic Surgery, Department of Surgery, Children's Hospital of Pittsburgh University of Pittsburgh Medical Center Pittsburgh Pennsylvania; ^2^ Division of Endocrinology and Diabetology, Department of Internal Medicine Medical University of Graz Graz Austria; ^3^ Department of Developmental Biology University of Pittsburgh Pittsburgh Pennsylvania; ^4^ University of Pittsburgh Cancer Institute Pittsburgh Pennsylvania

**Keywords:** diabetes, monocytes/macrophages, pancreatic cancer, pancreatitis, regeneration

## Abstract

**Introduction:**

Mounting evidence suggest that macrophages play crucial roles in disease and tissue regeneration. However, despite much efforts during the past decade, our knowledge about the extent of macrophages' contribution to adult pancreatic regeneration after injury or during pancreatic disease progression is still limited. Nevertheless, it is generally accepted that some macrophage features that normally would contribute to healing and regeneration may be detrimental in pancreatic cancer. Altogether, the current literature contains conflicting reports on whether macrophages act as friends or foe in these conditions.

**Methods and Results:**

In this review, we briefly review the origins of tissue resident and infiltrating macrophages and the importance of cellular crosstalking between macrophages and other resident cells in tissue regeneration. The primary objective of this review is to summarize our knowledge of the distinct roles of tissue resident and infiltrating macrophages, the impact of M1 and M2 macrophage phenotypes, and emerging evidence on macrophage crosstalking in pancreatic injury, regeneration, and disease.

**Conclusion:**

Macrophages are involved with various stages of pancreatic cancer development, pancreatitis, and diabetes. Elucidating their role in these conditions will aid the development of targeted therapeutic treatments.

## Graphical abstract

This is a review article on the role of macrophages in pancreatic regeneration and cancer.

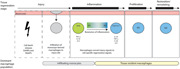

**Abbreviations**
ADAM17: disintegrin and metalloproteinase domain‐containing protein 17
ADAMTS1: disintegrin and metalloproteinase with thrombospondin motifs 1
Akt: v‐akt murine thymoma viral oncogene
BMP‐2: bone morphogenetic protein 2
CCR2: C‐C motif chemokine receptor 2
CREB: cAMP response element‐binding protein
CSC: cancer stem cell
CSF1: colony stimulating factor 1
CUX1: cut like homeobox gene
CXCL1: C‐X‐C motif ligand 1
CX_3_CR1: C‐X3‐C motif chemokine receptor 1
DAMP: damage‐associated molecular pattern
EGF: epidermal growth factor
EGFR: epidermal growth factor receptor
FoxO1: forkhead box O1 gene
HSC: hematopoietic stem cell
IAPP: islet amyloid polypeptide
ICAM‐1: intercellular adhesion molecule
IFN‐γ: interferon‐γ
IGF‐1: insulin‐like growth factor
IKK2/β: inhibitor of nuclear factor kappa B kinase subunit β
IL: interleukin
ISG15: IFN‐stimulated factor 15
LPS: lipopolysaccharide
MAPK: mitogen‐activated protein kinase
NLRP3: NOD‐, LRR‐ and pyrin domain‐containing protein 3
NF‐κB: nuclear factor kappa B
NGF: nerve growth factor
NO: nitric oxide
PAMP: pathogen‐associated molecular pattern
PanIN: pancreatic intraepithelial neoplasia
PDAC: pancreatic ductal adenocarcinoma
PDGF: platelet‐derived growth factor
PGE‐2: prostaglandin E2
pro‐NGF: pro‐nerve growth factor
PPAR‐γ: peroxisome proliferator‐activated receptor γ
RANTES: regulated upon activation, normal T cell expressed and presumably secreted
REG4: regenerating islet‐derived protein 4
Socs3: suppressor of cytokine signaling 3
STAT: signal transducer and activator of transcription
TAM: tumor‐associated macrophage
TGF: transforming growth factor
TGF‐β1: transforming growth factor‐β 1
Tie‐2: angiopoietin‐1 receptor
TLR: toll‐like receptor
TME: tumor microenvironment
TNF‐α: tumor necrosis factor‐α
VEGF: vascular endothelial growth factor

## 1 INTRODUCTION

There are multiple subpopulations of macrophages with distinct phenotypes dependent on the anatomical location and microenvironment stimuli. The term “macrophages” has been used to define a population of cells descended from a mononuclear lineage specialized for the identification, phagocytosis, and destruction of harmful biological agents, such as bacteria. However, growing evidence suggests that cellular crosstalk between macrophages and surrounding tissue resident cells is an important aspect of tissue regeneration. On the other hand, impaired macrophage function, either due to inherent defects or abnormal stimuli would instead contribute to disease progression. Here, we review an increasing body of literature that describe the involvement of macrophages during normal vs disease conditions.

## 2 MACROPHAGE ORIGINS

**Figure 1:**
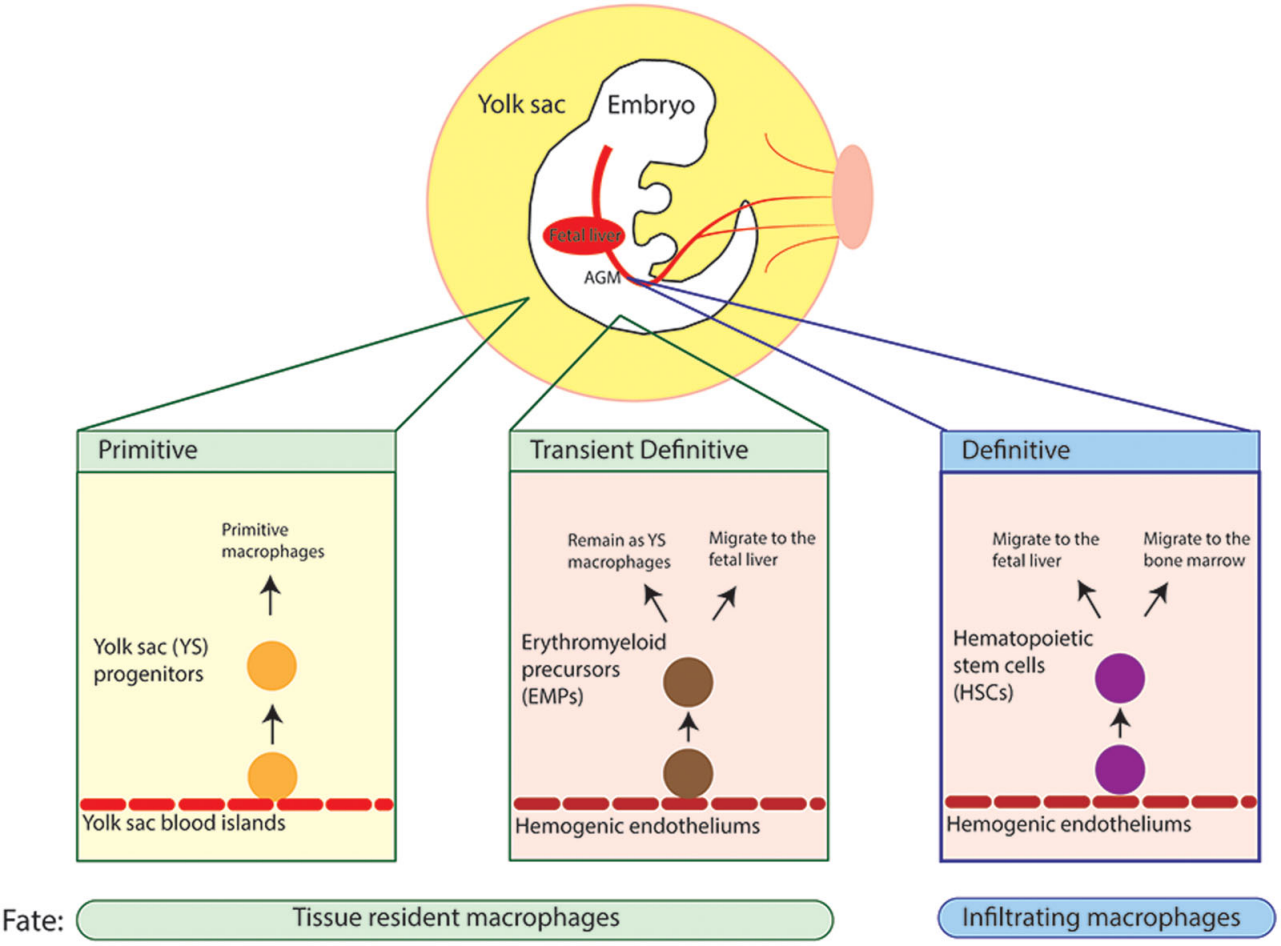
Fetal hematopoiesis. There are three waves of successive hematopoiesis that occur throughout development and adulthood: primitive, transient definitive, and definitive

Generally, there are three main populations of macrophages: yolk sac‐derived tissue resident macrophages, fetal liver‐derived tissue resident macrophages, and bone marrow–derived infiltrating macrophages (Figure 1). Mounting evidence indicates that tissue resident macrophages and infiltrating macrophages arise from three different waves of successive hematopoiesis that occur throughout development and adulthood. Tissue resident macrophages reside in the tissue and arise from the embryonic precursors generated through the first two waves of successive hematopoiesis originating in the extraembryonic yolk, whereas infiltrating macrophages arise from common myeloid progenitor cells generated during the third wave of hematopoiesis in the bone marrow.^1^, ^2^, ^3^, ^4^, ^5^, ^6^, ^7^, ^8^, ^9^, ^10^, ^11^ Briefly, the first wave is primitive hematopoiesis and it arises from the extraembryonic yolk sac and generates yolk sac progenitors that later become primitive macrophages, erythroblasts, and megakaryocytes.^10^, ^11^, ^12^, ^13^ The next wave is the transient definitive wave that produces erythromyeloid precursors^14^ that remain locally and become yolk sac macrophages or migrate to the fetal liver, upon establishment of fetal blood circulation, and differentiate into other cell lineages, such as monocytes.^11^, ^15^, ^16^ The third wave is definitive hematopoiesis. Immature hematopoietic stem cells (HSCs) emerge from the aorta‐gonad‐mesonephros and not only migrate to the fetal liver to mature into fetal HSCs, but also seed the fetal bone marrow to eventually generate adult HSCs.^10^, ^13^, ^15^, ^16^ The HSCs eventually produce discrete intermediate progenitors, like common myeloid progenitors that further differentiate into monocytes^17^ (Figure 1). As such, HSCs from the definitive wave are precursors to infiltrating macrophages; however, the identity of tissue resident macrophage precursors is still unclear. The current theories regarding the origins of tissue resident macrophages has been extensively discussed elsewhere.^11^

Tissues are able to replenish their population of resident macrophages through low‐level proliferation during steady‐state, but this can be increased with bone marrow–derived circulating monocytes that differentiate into macrophages during pathologies.^3^, ^9^, ^18^, ^19^, ^20^, ^21^ Several studies have shown that macrophage accumulation in tissue after injury is due to the recruitment of circulating monocytes, rather than the expansion of resident macrophages.^22^, ^23^, ^24^, ^25^, ^26^ Circulating monocytes are categorized into two groups based on expression of the Ly6C marker. Ly6C^+^ monocytes directly originate from the bone marrow progenitors, whereas Ly6C^−^ monocytes are derived from the Ly6C^+^ monocytes.^6^ The two types of blood circulating monocytes also have different functions. Ly6C^−^ monocytes patrol the vasculature to remove damaged endothelial cells. On the other hand, Ly6C^+^ monocytes in the vasculature sense tissue damage, infiltrate the injury site, and differentiate into macrophages.^27^, ^28^, ^29^ These infiltrating macrophages become polarized (or activated) in response to signals associated with pathogens or present in injured tissue. In fact, Infiltrating macrophages exist across a dynamic M1‐M2 polarization spectrum with an array of intermediate phenotypes in between.^30^ Stimulation from macrophage colony stimulating factor 1, interferon‐γ (IFN‐γ), and lipopolysaccharide (LPS) induces monocyte differentiation into M1‐like (classic) macrophages, whereas other factors such as interleukin‐4 (IL‐4), and IL‐13 induces monocyte differentiation into M2‐like (alternatively activated) macrophages.^19^, ^31^, ^32^, ^33^ M1 cells are implicated in initiating and sustaining inflammation through production of high levels of proinflammatory cytokines, reactive nitrogen and oxygen intermediates, while the more heterogeneous M2 cells are characterized by alternative arginine metabolism, exhibit a different chemokine expression profile and are associated with resolution or smoldering chronic inflammation.^32^, ^34^ In addition to many immune‐related cytokines, macrophages also produce numerous effector molecules such as platelet‐derived growth factor (PDGF), hepatocyte growth factor, fibroblast growth factor, transforming growth factor (TGF), and Wnt ligands.^35^ While macrophages can broadly be described as having an M1 or an M2 phenotype, it is important to keep in mind that these segregations were defined in vitro, under well‐defined stimuli and may not necessarily represent what happens in vivo. Several studies have shown the fate and phenotype of monocytes and macrophages are not as easily shaped by external stimuli as once believed. In addition to the Ly6C marker, monocytes can be further distinguished apart with the expression of surface receptors C‐C motif chemokine receptor 2 (CCR2) and C‐X3‐C motif chemokine receptor 1 (CX_3_CR1). Rodent studies have shown that CCR2^+^CX_3_CR1^+^Ly6C^hi^ and CCR2^−^CX_3_CR1^++^Ly6C^lo^ monocytes, upon appropriate stimulation, more readily and specifically differentiate into M1 and M2 macrophages, respectively.^36^, ^37^, ^38^, ^39^ Furthermore, macrophages may display the same M1 or M2 phenotype, but exhibit different expression profiles with respect for regenerative factors such as Wnt ligands or growth factors.^40^

**Figure 2:**
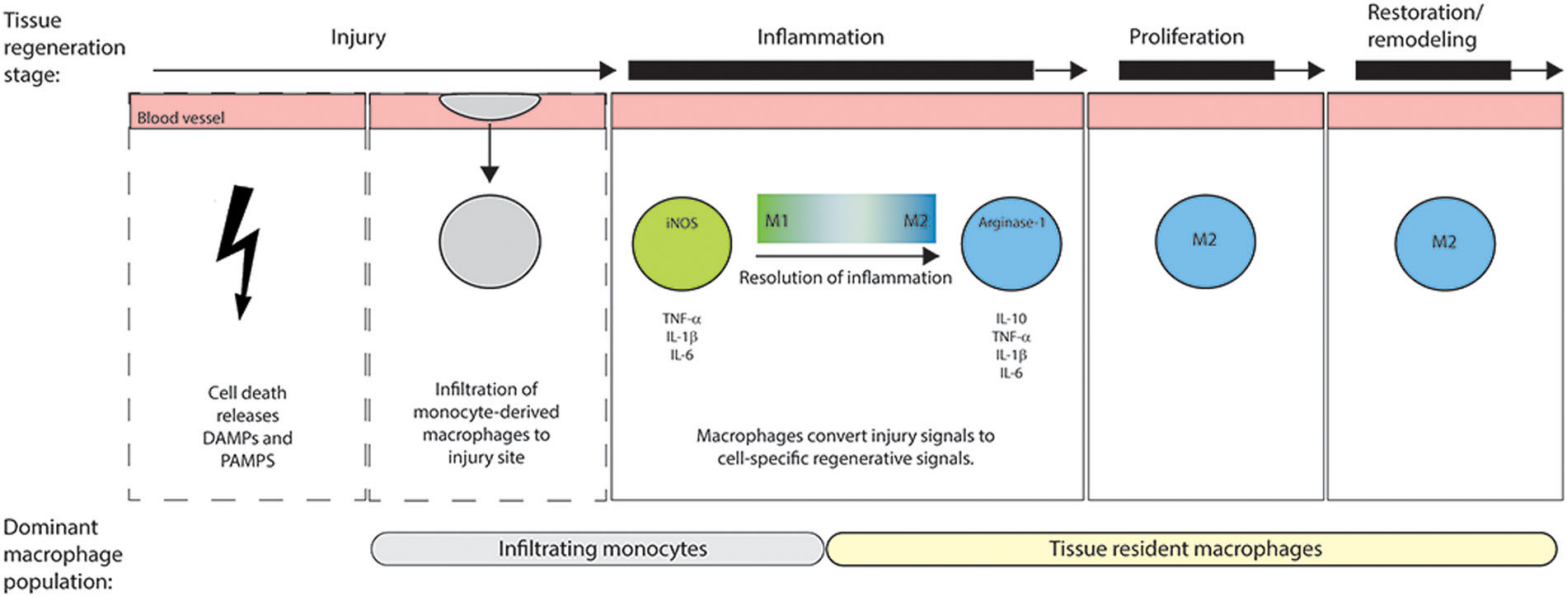
Tissue regeneration. Upon injury, monocyte‐derived macrophages are recruited to the injury site and stimulated to adopt a M1 phenotype. M1 macrophages are involved with clearing necrotic cells and tissue debris from the site, and inducing cytotoxic processes, initiate the inflammatory response and secrete pro‐inflammatory cytokines (e.g. TNF‐a, IL‐1b, and IL‐6). During the later stages of inflammation, there is a switch from the proinflammatory M1 to the prorepair M2 phenotype. M2 macrophages promote tissue repair through the secretion of proangiogenic and growth factors (e.g. IL‐10, TNF‐a, IL‐1b, and IL‐6) and stimulation of fibroblast deposition of granulation tissue. The macrophages predominantly involved with the initial inflammation response are monocyte‐derived macrophages, whereas macrophages involved in inflammation resolution and tissue repair are tissue resident macrophages in origin

Altogether, the current literature highlights the necessity of a revision in how we define different macrophage polarization phenotypes, at least in studies exploring the regenerative properties of macrophages.

## 3 MACROPHAGES AND TISSUE REGENERATION

Tissue regeneration is defined as the process in which damaged or diseased tissue is renewed and regrown or replaced, respectively. There are several types of tissue injury that can occur, such as pathogen entry, oxidative stress, or mechanical damage.^41^, ^42^ On the other hand, some tissues like the gut have constant low levels of inflammation to maintain intestinal homeostasis.^43^ Regardless of the type of insult, cell death induces the release of damage‐associated molecular patterns (DAMPs) or pathogen‐associated molecular patterns (PAMPs), which activate a number of receptors and release cytokines and chemokines to induce an inflammatory response and leukocyte recruitment to the site of injury.^41^, ^42^, ^44^ The initiated tissue regeneration process can be categorized into three different stages: inflammation, proliferation, and restoration/remodeling. A successful tissue regeneration process relies on a complex interaction between cells that provide necessary regenerative signals and cells that are receptive to those signals. Several studies have shown how macrophages provide and orchestrate the cues necessary for regeneration after injury.^22^, ^35^, ^45^, ^46^, ^47^, ^48^, ^49^, ^50^, ^51^, ^52^, ^53^, ^54^, ^55^ In general, infiltrating monocyte‐derived macrophages are more involved with the early inflammatory response, from clearing necrotic cells and tissue debris from the site, inducing cytotoxic processes, and promoting inflammation. On the other hand, tissue resident macrophages hold a greater role during inflammation resolution and tissue repair by producing proangiogenic and growth factors and stimulating fibroblast deposition of granulation tissue.^9^, ^41^, ^42^ Throughout tissue regeneration, there is a general pattern of monocyte‐derived macrophages infiltrating the injury site, adopting a proinflammatory M1 phenotype in the early stages, and shifting to an anti‐inflammatory, prorepair M2 phenotype in the later stages^56^, ^57^ (Figure 2). The role of M1 and M2 macrophages in tissue regeneration of liver, skeletal muscle, kidney, and nerves are reviewed in depth elsewhere.^56^

The sequential occurrence of M1 and M2 macrophage phenotypes is crucial for normal tissue regeneration^57^; however, the mechanism underlying the phenotypic switch is unclear. One theory is that the microenvironment of some tissues, like the central nervous system, liver, skeletal muscle, heart, and pancreas, produce temporally dynamic and transient signals that induce in situ M1 to M2 macrophage phenotype conversion. An in situ conversion would explain why infiltrating macrophages are seen to possess both proinflammatory and anti‐inflammatory/prorepair roles.^22^, ^58^, ^59^, ^60^, ^61^, ^62^, ^63^, ^64^ Signals that induce an in situ conversion can be an increase/decrease in specific factors, activation of a signaling pathway, or crosstalk between macrophages and other cells. To begin with, a reduction in DAMPs, PAMPs, and apoptotic neutrophils has been shown to induce the switch in macrophage phenotype from proinflammatory to anti‐inflammatory.^41^ In addition, during skeletal muscle regeneration, the phagocytosis of cellular debris and the expression of secretory leukocyte peptidase inhibitor and peroxisome proliferator‐activated receptor γ (PPAR‐γ) promote conversion of inflammatory CX_3_CR1^lo^/Ly6C^+^ macrophages into anti‐inflammatory CX_3_CR1^hi^/Ly6C^−^ macrophages. CX_3_CR1^hi^/Ly6C^−^ macrophages promote myogenic differentiation and myofiber growth and protection of myotube differentiation, which is crucial for proper fiber membrane repair.^22^ Moreover, in the kidney, the Wnt/β‐catenin signaling pathway via Wnt3a‐induced upregulation and activation of signal transducer and activator of transcription 3 (STAT3) has been shown to induce M2 macrophage polarization.^65^ Finally, there is evidence of crosstalk playing a role in the phenotypic switch in macrophages during tissue regeneration. In the heart, the switch from inflammatory to anti‐inflammatory macrophages is mediated by bone marrow–derived mesenchymal stromal cells via IL‐10 secretion.^66^, ^67^ However, an alternative theory is that there is a sequential recruitment of macrophages and expression of specific cell surface receptors at the injury site. A study on myocardial infarction in mice showed that the expansion of circulating Ly6C^hi^ monocytes and expression of receptor CCR2 led to the migration of proinflammatory Ly6C^hi^ monocytes to the injury site. Later, expression of receptor CX_3_CR1 led to the preferential recruitment of circulating Ly6C^lo^ monocytes to the site. Anti‐inflammatory Ly6C^lo^ monocytes express vascular endothelial growth factor (VEGF) and begin granulation tissue formation. Therefore, it is the expansion of circulating monocytes and specific CCR2 or CX_3_CR1 receptor expression that drives a preferential recruitment and phenotype of infiltrating macrophages, respectively.^47^

All in all, macrophages play numerous and crucial roles during tissue regeneration. Upon injury, monocyte‐derived macrophages infiltrate the site and the predominant phenotype is M1, which secrete proinflammatory factors to activate various signaling pathways. At some point, the dominant macrophage phenotype switches to M2, which are anti‐inflammatory and prorepair. There is a dispute over the mechanism behind the phenotypic switch; it could either be driven by an in situ conversion of M1 to M2 or a selective, sequential recruitment of macrophages. The monocyte‐derived macrophage populations are part of the initial inflammatory response, whereas tissue resident macrophages appear to be more involved with the resolution of inflammation and tissue repair.

## 4 MACROPHAGES CROSSTALK DURING TISSUE REGENERATION

Cellular crosstalk describes how one or more components of a signaling pathway affects another signaling pathway either directly or indirectly to produce a specific biological response.^68^ Several studies have shown that macrophages crosstalk with endothelial cells, smooth muscle cells, mesenchymal stem cells, and stellate cells to support different aspects of tissue regeneration, such as macrophage recruitment, macrophage polarization, cell proliferation, and angiogenesis. For example, macrophages express Wnt ligands and respond to Wnt signaling to promote endothelial cell proliferation and migration, which is important for angiogenesis.^69^ In particular, proinflammatory factors IFN‐γ and LPS upregulate Wnt5a expression in macrophages.^70^ Wnt5a is then able to regulate angiogenesis at multiple levels, which in turn can directly induce the expression of additional proangiogenic cytokines such as IL‐6, IL‐8, and IL‐1β.^71^, ^72^, ^73^, ^74^ Wnt5a also indirectly induces the proliferation and migration of endothelial cells and increase in Tie‐2 expression in endothelial cells and macrophages.^69^, ^75^ Macrophages are then further stimulated by angiopoietin 2 to become proangiogenic.^76^ In addition, Wnt5a upregulates CCL2 expression in endothelial cells to indirectly recruit more macrophages.^77^ Moreover, the crosstalk between macrophages and smooth muscle cells promotes angiogenesis during atherogenesis. Coculture of smooth muscle cells and macrophages lead to an increase in tumor necrosis factor‐α (TNF‐α), IL‐6, IL‐1β, and toll‐like receptor 2 (TLR2) levels, which altogether increases secretion of angiogenic factors VEGF and IL‐8 likely through TLR signaling pathways.^78^ Another study found that transforming growth factor‐β1 (TGF‐β1) can also stimulate VEGF production and angiogenesis via the TGF‐β signaling pathway.^79^ Furthermore, the crosstalk between macrophages and mesenchymal stem cells is crucial for bone healing.^80^, ^81^, ^82^ Macrophages release chemokines (CCL2, stromal cell‐derived factor 1, C‐X‐C motif ligand 8 [CXCL8)], proinflammatory cytokines (TNF‐ɑ, IL‐1β, IL‐6), and osteoinductive factors (oncostatin M, bone morphogenetic protein 2, prostaglandin E2 [PGE‐2]) which are received by mesenchymal cells. In response to these signals, mesenchymal cells direct osteoprogenitor differentiation into osteoblasts, and release immunosuppressive signals, like CCL2, VEGF‐A, PGE‐2, and nitric oxide (NO), that regulate macrophage recruitment and activity.^82^, ^83^ In addition, the crosstalk between macrophages and hepatic stellate cells, a liver‐specific pericyte that behaves like mesenchymal stem cells, is important for macrophage polarization and progression of liver repair.^84^, ^85^, ^86^, ^87^, ^88^ Following liver injury, injured parenchymal cells release PAMPs and/or DAMPs, which bind to TLR4 receptors on hepatic stellate cells or other pericytes. Hepatic stellate cells secrete chemoattractants like macrophage migration inhibitory factor, CXCL1, CCL2, IL‐6, and IL‐8, to recruit neutrophils and monocytes to the site.^89^, ^90^, ^91^, ^92^, ^93^, ^94^, ^95^ Monocyte‐derived M1 macrophages and neutrophils secrete amphiregulin and IL‐17a, respectively, and these two proteins convert TGF‐β to its active form so it can activate hepatic stellate cells and pericytes.^96^, ^97^, ^98^, ^99^, ^100^ Over time the number of activated pericytes and activated TGF‐β levels increase. Increasing levels of TGF‐β stimulate a M1 to M2 polarization shift via Akt/SNAIL and Akt/FoxO1 signaling pathways, and secretion of MMP‐7 by scar‐associated macrophages to promote differentiation of hepatocyte‐produced pro‐NGF into nerve growth factor (NGF). NGF binds to p75 NGF receptor on activated pericytes and induces apoptosis and fibrosis.^101^, ^102^, ^103^, ^104^, ^105^, ^106^, ^107^ Furthermore, macrophages crosstalk with satellite cells to regulate the myogenesis process.^108^ Not only do macrophages secrete proinflammatory cytokines, such as IL‐6, TNF‐α, and PGE‐2, but also the enzyme ADAMTS1, which induces satellite cell activation and proliferation and promotes muscle regeneration.^60^, ^61^, ^109^ Moreover, anti‐inflammatory macrophages secrete cytokines, such as IL‐4 and insulin‐like growth factor 1, that induces myoblast differentiation and myofiber growth.^41^, ^108^

To sum up, there is increasing evidence that crosstalk between macrophages and other resident cells help drive tissue regeneration.

## 5 MACROPHAGES IN THE REGENERATING PANCREAS

**Figure 3:**
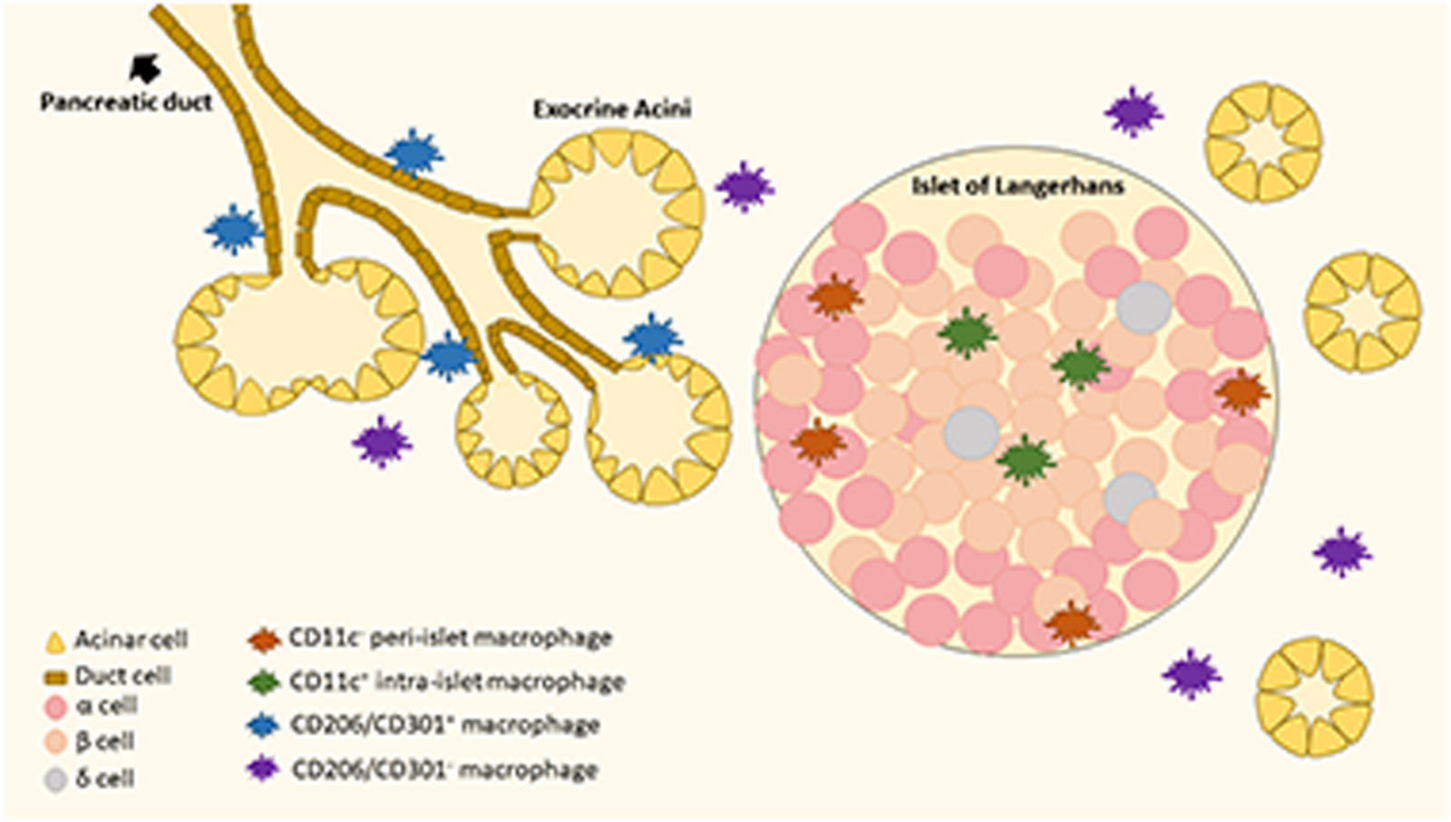
Diagram of proposed tissue resident macrophages in the pancreas: CD11c^−^ [R1] peri‐islet (orange), CD11c^+^ [R2] intraislet (green), CD206/CD301^+^ (blue), and CD206/CD301^−^ (purple)

The origins and phenotypes of resident macrophages differ within the intrapancreatic microenvironment. One study describes a single population of resident macrophages residing in the islets of Langerhans that arose from definitive hematopoiesis, remained locally in the islets since birth, proliferate in situ slowly, and display the M1 phenotype.^110^ However, another study found two separate subsets of resident macrophages, using surface markers CD11c and F4/80, residing within the islets. CD11c^−^ [R1] and CD11c^+^ [R2] resident macrophages reside in the peri‐ and intraislets, respectively, and are not derived from circulating monocytes.^111^ There are two populations of resident macrophages in the interacinar stroma, which are distinguished apart by CD206 (mannose receptor) and CD301 (CLEC10A) expression. The CD206/CD301^+^ resident macrophages arise from primitive hematopoiesis, concentrate around the pancreatic ducts, and display an M2 phenotype. On th eother hand, the CD206/CD301^−^ resident macrophages arise from definitive hematopoiesis, replenished by circulating monocytes, and display an M2 phenotype (Figure 3).^110^

**Figure 4:**
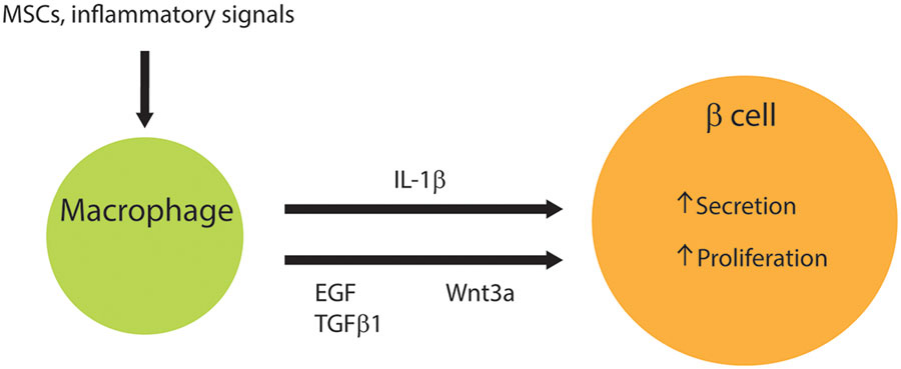
M2 macrophages crosstalk with β cells to promote β‐cell proliferation and insulin secretion. EGF, epidermal growth factor; IL‐1β, interleukin‐1β; TGF‐β1, transforming growth factor‐β1

Since fetal macrophages are thought to contribute to pancreatic islet morphogenesis and remodeling during the fetal and neonatal stages, it is also possible that macrophages play a role in adult pancreatic regeneration.^112^, ^113^, ^114^ In fact, there is emerging evidence showing that macrophages crosstalk with resident cells via release of factors like IL‐1β, epidermal growth factor (EGF), TGF‐β1, and Wnt3a to promote β‐cell proliferation and pancreas regeneration.^55^, ^115^, ^116^, ^117^, ^118^ To begin with, islet macrophages release low levels of IL‐1β, which enhances β‐cell insulin secretion especially during periods of acute stress.^119^ In addition, macrophage crosstalk with β‐cells assist in β‐cell proliferation and regeneration following pancreatic injury. During the inflammation resolution phase, M2 macrophages release EGF and TGF‐β1 and these signals are received by β‐cells. EGF acts on epidermal growth factor receptor (EGFR) to inhibit SMAD2 nuclear translocation, thereby inhibiting TGF‐β signaling. TGF‐β1 acts on TGF‐β receptor to upregulate SMAD7, which not only causes nuclear exclusion of cell cycle regulator p27 but also increases cell cycle activators cyclin D1 and cyclin D2. Altogether, this promotes β‐cell proliferation.^120^, ^121^ Moreover, in a diabetic phenotype mouse model, M2 macrophages release Wnt3a and this signal is received by receptors at the surface of β‐cells. This leads to inhibition of glycogen synthase kinase 3 and subsequent β‐catenin translocation into the nucleus. There is also a marked increase in cyclin D2 in the nucleus. Altogether, the activation of the β‐cell Wnt/β‐catenin signaling pathway promotes β‐cell replication.^122^ Moreover, in a mouse model with VEGF‐A overexpression‐induced β‐cell loss, macrophage crosstalk with islet endothelial cells was important for β‐cell proliferation.^123^ These reports indicate M2 macrophages crosstalk with multiple cell types in islets is important to β‐cell regeneration following pancreatic injury (Figure 4). Furthermore, the origins of M2 macrophages responsible for β‐cell proliferation have been identified as resident tissue macrophages.^63^ In fact, both the peri‐ and intraislets resident macrophages are found to promote β‐cell proliferation.^111^

## 6 MACROPHAGES IN PANCREATIC DISEASE

### 6.1 Diabetes

There is much evidence showing that prolonged macrophage activation causes β‐cell death during type 1 and type 2 diabetes mellitus via the release of cytokines and nutrients, respectively. The onset of type 1 diabetes is marked by the infiltration of macrophages and T cells in the islets of Langerhans.^124^, ^125^, ^126^ In type 1 diabetes, macrophage secretion of cytokines IL‐1β and/or TNF‐α + IFN‐γ activates nuclear factor kappa B (NF‐κB) and STAT1.^127^, ^128^, ^129^ NF‐κB activation then leads to NO production and chemokines as well as endoplasmic reticulum (ER) calcium production. ER stress and mitochondrial death signals subsequently induce β‐cell death.^129^

On the other hand, β‐cell death in type 2 diabetes is caused by chronic elevation of glucose and free fatty acid levels and crosstalk with islet M1 macrophages.^116^, ^117^, ^129^ The free fatty acids force β cells to produce islet amyloid polypeptide (IAPP). The release of IAPP and adenosine triphosphate from β cells signals for the recruitment of bone marrow–derived monocytes and M1 macrophage accumulation to the islets. The elevated levels of glucose lead to activation of the NLRP3‐dependent inflammasomes and processing and production of proinflammatory factor IL‐1β in M1 macrophages. M1 macrophages also release TNF‐α and the increased levels of both TNF‐α and IL‐1β crosstalk with β cells and cause β‐cell dysfunction and eventual cell death. β‐cell death releases more chemokines and cytokines, thereby creating a feedback loop that continues to drive inflammation and the β‐cell failure that is characteristic of type 2 diabetes.^116^, ^117^

Although there is significant evidence that shows macrophages cause β‐cell death during type 1 and type 2 diabetes, there is evidence that suggests that macrophages are also involved with pancreas and β‐cell regeneration.

### 6.2 Pancreatitis

In the healthy pancreas, acinar cells and ductal cells are responsible for the production of digestive enzymes and transportation of enzymes to the gut, respectively. During acute pancreatitis, there is the premature activation of trypsinogen in acinar cells, leading to autodigestion of the pancreas, stimulation of M1 macrophages, and release of proinflammatory factors. Proinflammatory factors TNF‐α, IL‐1β, IL‐6, and monocyte chemoattractant protein 1 can recruit neutrophils and more monocytes to the pancreas and increase the release of proinflammatory mediators.^130^, ^131^ More importantly, the release of cytokines TNF‐α and RANTES by macrophages stimulates the activation of NF‐κB in acinar cells to induce acinar cell transdifferentiation into a duct‐like progenitor cell types, which is termed acinar‐to‐ductal metaplasia (ADM).^131^, ^132^ Notch receptors^133^, ^134^ and EGFR^135^, ^136^ have also been shown to induce ADM in acinar cells. M2 macrophages are recruited to help the cells revert back to acinar cells and initiate tissue remodeling and repair.^136^, ^137^, ^138^, ^139^ Ultimately, ADM decreases the production of digestive enzymes, mitigates the inflammatory response, and sets the stage for tissue repair. Recurrent pancreatic injury can eventually lead to the development of chronic pancreatitis, which is characterized by chronic inflammation, irreversible fibrosis, acinar cell atrophy and contorted ducts.^140^ Unlike acute pancreatitis, most macrophages in chronic pancreatitis display a M2 phenotype.^141^ The infiltrating macrophages crosstalk with and activate nearby pancreatic stellate cells (PSCs) via the TGF‐β/PDGF signaling pathway. PSCs are periacinar stromal cells, which are present in a quiescent state in healthy pancreas. PSCs are activated during initial phases of pancreatic injury and play a key role in pancreatitis and pancreatic cancer as the predominant source of collagen in the fibrotic pancreas. Activated PSCs release IL‐4 and IL‐13, which promotes M2 macrophage polarization. In fact, inhibition of IL‐4 and IL‐13 in ex vivo human tissue and mice has been shown to lead to a decrease in M2 macrophages levels and fibrosis progression.^141^ Most notably, chronic pancreatitis is strongly associated with the development of pancreatic ductal adenocarcinoma (PDAC).^142^

To sum up, there is a heterogenous population of macrophages in the pancreas that differ in origin and function. Recent studies show that macrophages crosstalk with other cells present in the pancreas to promote β‐cell proliferation and pancreatic regeneration following injury. M1 macrophages play a dominate role in resolving acute pancreatitis and M2 macrophages play a dominate role in the chronic pancreatitis and β‐cell proliferation.

### 6.3 Pancreatic cancer

Patients with pancreatic cancer have a poor prognosis and a survival rate of about 6% within 5 years of initial diagnosis. The most common type of pancreatic cancer is PDAC.^143^ The late detection and aggressive nature of PDAC makes it notoriously difficult to treat, therefore, understanding the drivers of PDAC initiation, progression, and metastasis is imperative to developing therapy treatments.^144^ Nearly 95% of all pancreatic cancers have one of three different proto‐oncogene mutations of Kras within the pancreatic acinar cells.^145^, ^146^, ^147^ Therefore, the general steps toward the development of PDAC is as follows: acquirement of a Kras mutation in an acinar cell, ADM, formation of pancreatic intraepithelial neoplasia (PanIN) lesions or other lesions, progression from lesion to PDAC, and continued promotion of tumor growth and metastasis. Interestingly, macrophages have been shown to play a role at each of these steps toward PDAC. Inflammatory macrophages promote the formation of precursor PanIN lesions or other lesions by crosstalking with acinar cells carrying a Kras mutation to induce inappropriate activation of signaling pathways that bring on ADM, and secreting factors that promote tissue remodeling. Alternatively activated macrophages drive the development of PanIN lesions to PDAC. In addition, alternatively activated tumor‐associated macrophages (TAMs) promote further tumor growth and PDAC metastasis. We do acknowledge that there is still an ongoing debate on which cells contribute to the pancreatic cancer, and while other cell types such as duct cell cannot be ruled out, the current review focuses on acinar origin of the PDAC.

To begin with, inflammatory M1 macrophages are stimulated by Kras^G12D^‐acinar cells to release factors that cause inappropriate activation of signaling pathways, such as the NF‐κB, Notch, EGFR/mitogen‐activated protein kinase (MAPK), Wnt/β‐catenin, and STAT3/suppressor of cytokine signaling 3 (Socs3), to stimulate ADM.^148^ Acinar cells with the Kras^G12D^ mutation upregulate ICAM‐1 expression and a fraction of it is shed as a soluble form (sICAM‐1), which acts as a chemoattractant and recruits M1 macrophages. The M1 macrophage interacts with the Kras^G12D^‐acinar cell and secretes MMPs, like MMP‐9, to degrade the extracellular matrix and promoting tissue remodeling. M1 macrophages also secrete inflammatory cytokines, such as TNF, that induce activation of the NF‐κB signaling pathway to drive ADM in acinar cells.^131^, ^145^ The Kras^G12D^ mutation stimulates production of activator protein 1 (AP1), which induces IL‐1a overexpression. IL‐1a activates downstream inhibitor of nuclear factor kappa B kinase subunit β (IKK2/β), which then activates NF‐κB. NF‐κB promotes transcription of IL‐1a and p62, and these two factors act on IKK2/β to establish an autoregulatory feedback loop for constitutive activation of the NF‐κB signaling pathway.^149^ In addition, there is crosstalk between the NF‐κB and Notch signaling pathways in Kras^G12D^‐acinar cells. IKK2 from the NF‐κB pathway synergizes with basal Notch to transcribe Notch target genes, one being a suppressor of anti‐inflammatory transcription factor PPAR‐γ. As such, this helps to maintain the inflammatory response initiated by Kras^G12D^‐acinar cells.^150^ Moreover, the Kras^G12D^ mutation activates the MAPK signaling pathway. Kras upregulates EGFR expression and enhances EGFR activity via EGFR ligand sheddase, ADAM17.^151^ An example of crosstalk between TAM and the epithelium is the stimulation of EGFR expression in neoplastic epithelium by macrophages and stimulation of macrophages to secrete EGFR ligands by Kras^G12D^‐acinar cells.^136^, ^152^ Some ligands bind to EGFR on Kras^G12D^‐acinar cells and activate the MAPK signaling pathway and repress acinar‐specific transcription factors,^136^, ^153^, ^154^ whereas other ligands stimulate stromal fibroblasts to produce collagen.^136^ Interestingly, low levels of Wnt signaling in cell lines with Kras mutations crosstalk with the MAPK signaling pathway to also promote ADM and PanIN formation.^146^ Furthermore, myeloid cell secretion of IL‐6 induces the activation of the STAT3/Socs3 signaling pathway in Kras^G12D^‐acinar cells. The signaling pathway is continuously activated in a feed‐forward response loop.^155^ STAT3 signaling has also been shown to help Kras^G12D^‐acinar cells maintain a proliferative, dedifferentiated state and contribute to inflammation. IL‐6 is not the only pathway that promotes STAT3 activation. It was recently reported that extracellular high mobility group box 1, either passively released by damaged/dying neoplastic cells, or actively secreted by TAMs stimulates prolactin expression by macrophages. The macrophage‐derived prolactin binds to its cognate receptor on PanIN cells, where it maintains focal adhesion kinase 1 and STAT3 activity. In addition, prolactin may promote fibrosis through PRLR‐expressing resident macrophages.^156^

**Figure 5:**
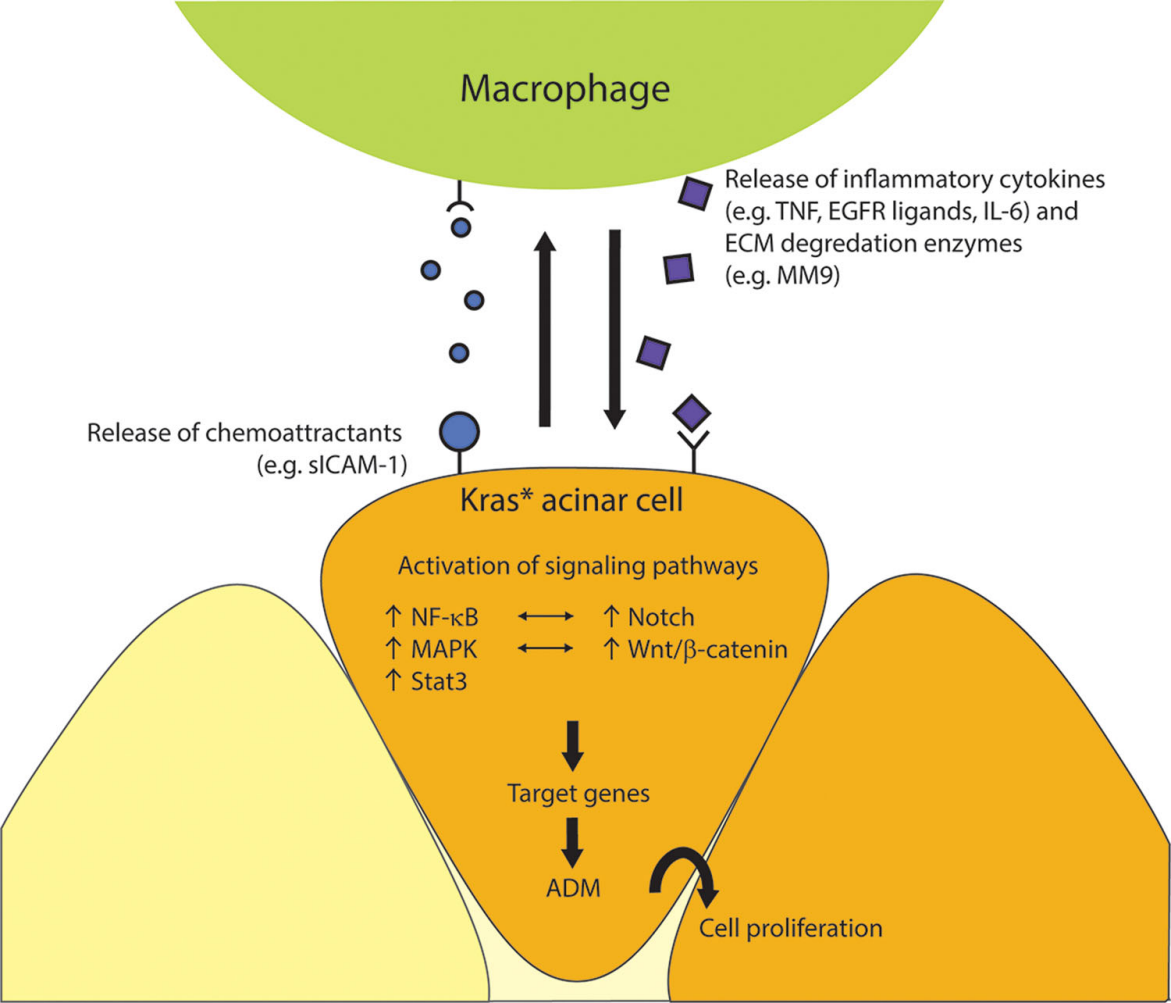
Schematic of proposed macrophage crosstalk and subsequent signaling cascades initiated by the Kras mutation (Kras*) in an acinar cell to induce acinar‐to‐ductal metaplasia (ADM). The Kras* acinar cell recruits M1 macrophages through the release of chemoattractants (blue circles). The macrophage subsequently releases proinflammatory cytokines (purple triangles) that activate various signaling pathways, such as nuclear factor kappa B (NF‐κB), Notch, mitogen‐activated protein kinase (MAPK), Wnt/β‐catenin, and STAT3/Socs3, in acinar cells. The activation of these signaling pathways leads to the transcription of target genes that ultimately suppress acinar‐specific transcription factors and/or anti‐inflammatory transcription factors, thereby inducing ADM. The NF‐κB and Notch signaling pathways and the MAPK and Wnt/β‐catenin signaling pathways also participate in crosstalk. Macrophages also secrete extracellular matrix (ECM) degradation enzymes, which promotes tissue remodeling. The combined actions of irreversible ADM, continuous macrophage secretion of inflammatory cytokines and ECM degradation enzymes, and sustained cell proliferation is permissive for precursor intraepithelial neoplastic lesions (PanINs) or other lesions formation. Socs3, suppressor of cytokine signaling 3; STAT3, signal transducer and activator of transcription 3

In addition, Stat3 signaling controls MMP‐7 expression, which regulates tumor size and metastasis, in Kras^G12D^‐acinar cells.^157^ Ultimately, the Kras^G12D^ mutation continuously promotes crosstalking with macrophages that secrete factors that activate signaling pathways in acinar cells that not only induces ADM but also maintains the dedifferentiated cellular state.^148^ In addition, the Kras^G12D^ mutation sustains, rather than directly causes, cell proliferation,^158^ thereby allowing for the formation of intraepithelial neoplastic lesions (PanINs) or other lesions (Figure 5).^136^, ^145^

**Figure 6:**
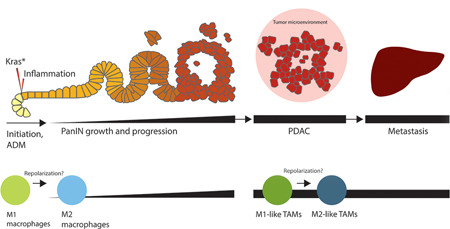
Diagram of the appearance of macrophages during the development and progression of pancreatic ductal adenocarcinoma (PDAC). Inflammatory M1 macrophages are stimulated by acinar cells carrying a Kras mutation (Kras*) to release factors that activate various signaling pathways to induce acinar‐to‐ductal metaplasia (ADM). Inflammatory macrophages undergo repolarization and become alternatively activated M2 macrophages. Without Kras*, M2 macrophages would assist with the redifferentiation of acinar cells and initiate tissue remodeling and repair. However, with Kras*, factors secreted by the M2 macrophages drive PanIN growth and progression instead. Ultimately, PDAC develops and the tumor‐associated macrophages (TAMs) in the tumor microenvironment (TME) promote tumor growth, progression, and metastases of PDAC. M1‐like TAMs are observed in the beginning stages of PDAC and are involved with inflammation. On the other hand, M2‐like TAMs are in greater abundance during the later stages of PDAC and reinforce tumor growth, invasion, vascularization, and metastasis

Next, the role of macrophages during the progression from PanIN lesion to PDAC is still largely unknown. However, a recent study shows that the inflammatory M1‐like macrophages switch to tumor‐promoting, alternatively activated M2‐like macrophages at ADM/PanIN lesions (Figure 6). In particular, IL‐13, which is likely produced by PSCs, binds to receptor IL‐13‐Rα1 on inflammatory macrophages to initiate the polarization switch towards an alternatively active macrophage. Alternatively activated macrophages then secrete factors such as CCL2 and IL‐1ra to drive fibrogenesis and PanIN lesion growth.^159^

Lastly, circulating monocytes and macrophages are recruited to the stromal compartment of the tumor microenvironment (TME), henceforth known as TAMs, where they secrete factors to alter the TME and drive PDAC progression and metastasis.^160^ The stroma is comprised of many different cell types,^161^ but this review will only focus on TAMs. TAMs found in PDAC can be either resident macrophages or monocyte‐derived macrophages in origin. Resident‐derived TAMs undergo in situ proliferation and are responsible for the fibrotic response and tumor progression, whereas monocyte‐derived TAMs help with antigen presentation and immune response.^160^ All tumors have M1‐like and M2‐like TAMs, but the amount of each phenotype changes over the course of tumorigenesis.^162^, ^163^ During the beginning stages of PDAC, M1‐like macrophages are found in abundance at sites of chronic inflammation and tumors. As tumor invasion and vascularization begins, the macrophages switch to the M2‐like phenotype (Figure 6).^164^, ^165^ Most TAMs have a M2‐like phenotype and their presence in PDAC is associated with poor outcomes.^166^, ^167^ The mechanism behind TAM polarization to an M2‐like phenotype is still unclear, but there is growing evidence that the activation of signaling pathways via crosstalk can induce repolarization. In cell lines, cancer cells secrete regenerating islet‐derived regenerating islet‐derived protein 4 (REG4). REG4 activates the EGFR/AKT/cAMP response element‐binding protein signaling pathway and this may, in part, induce M2 polarization.^168^ In addition, in cell lines, tumor and other stromal cells secrete TGF‐β, which induces homeobox transcription factor CUX1 and the NF‐κB signaling pathway via acetylation, thereby repressing NF‐κB‐regulated proinflammatory cytokines and antagonizing TAM M1‐like phenotype.^169^ Furthermore, crosstalk between pancreatic tumor cells and macrophages via exosomal miRNA is thought to also induce M2 polarization. The hypoxic microenvironment of solid tumors promotes pancreatic tumor cells to release miR‐301a‐3p‐rich exosomes. These exosomes activates the phosphatase and tensin homolog/PI3Kγ pathway to induce M2 macrophage polarization.^170^ Crosstalk with cancer stem cells (CSCs) have also been shown to induce an M2‐like phenotype in macrophages via Nodal/Activin A and TGF‐β1 secretion and activation of STAT3 signaling.^171^, ^172^ Ultimately, the M2‐like TAMs are responsible for promoting PDAC tumorigenesis and metastasis.

Interestingly, TAMs create an immunosuppressive microenvironment early on in tumorigenesis, hence the disabled cancer immunosurveillance system allows for unhindered disease progression.^173^ It is hypothesized that TAMs inhibit natural killer T cell response by: (a) depleting the pool of metabolites necessary for T cell proliferation, (b) producing anti‐inflammatory cytokines, (c) inducing expression of inhibitory receptors and other immune checkpoint ligands, and/or (d) producing chemokines and cytokines that recruit and sustain, respectively, Tregs in the TME.^174^ Moreover, TAM secretion of cytokines and factors, activation of various molecular signaling pathways, and subsequent promotion of cancer cell proliferation, angiogenesis, vascularization, and matrix remodeling is well‐known and extensively covered in other reviews.^163^, ^175^, ^176^, ^177^ Furthermore, there is emerging evidence that shows cellular crosstalking plays a role in PDAC progression.^178^, ^179^, ^180^ In particular, crosstalk between macrophages and CSCs enhances tumorigenesis.^181^ For example, reduction in the number of infiltrating macrophages resulted in a significant decline of CSCs. In addition to controlling the number of CSCs, TAM can also increase the tumor‐initiating capacity of CSCs through STAT3 signaling pathway.^182^ In vitro primary human pancreatic cancer spheres show CSCs secrete IFN‐β and, in turn, stimulates TAMs to secrete IFN‐stimulated factor 15 (ISG15). ISG15 is shown to enhance CSC self‐renewal and tumorigenic properties.^183^ Moreover, polarized TAMs are also shown to secrete antimicrobial peptide human cathelicidin 18/LL‐37, which binds to CSC receptors and enhance their stemness and tumorigenic properties.^172^ Finally, binding of CD47, which is expressed by CSCs, to SIRPα on macrophages leads to prevention of CSCs phagocytosis by macrophages.^184^ Altogether, TAM‐mediated paracrine signaling promotes the stem‐like features of CSCs, thereby enhancing tumor progression, metastasis, and chemoresistance in PDAC. Interestingly, there is also evidence that liver macrophages play a role in promoting pancreatic cancer‐related illnesses, such as cachexia (wasting syndrome). In PDAC patients, there is an increase in peripheral blood mononuclear cells.^185^, ^186^, ^187^ It is hypothesized that the monocytes infiltrate the liver, triggering activation of liver parenchymal cells, and inducing the release of proinflammatory cytokines, like TNF‐α, IL‐6, and IL‐8.^187^, ^188^, ^189^, ^190^ In turn, this activates NF‐κB and STAT3 transcription factors and hepatocytes for additional release of proinflammatory cytokines.^190^

In summary, the Kras mutation and inflammation activates additional signaling pathways that not only induces ADM, but also prevents redifferentiation back to acinar cells and subsequently leads to the formation of precursor PanIN lesions. In the ADM/PanIN lesions, macrophages undergo a phenotypic switch from inflammatory to alternatively activated. The alternatively activated macrophages continue to drive the progression from PanIN lesion to PDAC. M2‐like TAMs continue to drive tumor growth, progression, and metastases of PDAC via activation of various molecular signaling pathways. Notably, there is emerging evidence that shows crosstalk between macrophages and CSCs in the TME play a role in supporting tumorigenesis and metastasis of PDAC. Given the involvement of macrophages in general, and M2 macrophages in particular in various stages of PDAC development, targeted therapeutic treatments aiming to reduce the number of M2 subtype either through inhibition of recruitment, specific ablation or conversion to M1 phenotype has shown promising results in numerous clinical trials.^191^

## 7 CONCLUSION

Macrophages are indispensable not only for fighting harmful biological agents but also for healing and tissue regeneration. In that regard, M1 and M2 phenotypes play separate, yet equally important roles. Macrophages are like good Samaritans, always ready to help, but sometimes they unwillingly help the wrong side. In other words, they are villains by circumstances, not necessarily by actions.

## CONFLICT OF INTERESTS

The authors declare that there are no conflict of interests.

## Data Availability

Data sharing is not applicable to this article as no new data were created or analyzed in this study.
